# The Need for Health Systems to Engage With and Support Youth who are Caregivers—A Lived Experience Perspective From Young Carers

**DOI:** 10.1177/23743735251383246

**Published:** 2025-10-06

**Authors:** Alexandre Grant, Nicholas Goberdhan, Kristie Mar, Amanda Ramkishun, Samiha Rahman, Tyler Redublo, Isabelle Caven, Karen Okrainec

**Affiliations:** 1 Independent Advisor, 7989 University Health Network, Toronto, ON, Canada; 2 12357 University of Alberta Faculty of Medicine & Dentistry, Edmonton, AB, Canada; 3 Department of Communications Studies, 5618 Concordia University, Montreal, QC, Canada; 4 12358 University of British Columbia Faculty of Medicine, Vancouver, BC, Canada; 5 600011 Centre for Aging + Brain Health Innovation, Toronto, ON, Canada; 6 Translational Research Program, 12366 Temerty Faculty of Medicine, University of Toronto, ON, Canada; 7 Toronto General Hospital Research Institute, 7989 University Health Network, Toronto, ON, Canada; 8 Department of Medicine, 206712 University of Toronto, ON, Canada; 9 Institute of Health Policy, Management, and Evaluation, 233846 University of Toronto, ON, Canada

**Keywords:** caregiving, young carers, access to care, clinician–patient relationship, healthcare planning/policy, patient perspectives/narratives

## Abstract

Caregivers under the age of 25, or young carers, lack significant recognition and support across sectors of education, employment, and healthcare. As young carer advisors on a prior research project exploring Canadian healthcare providers’ awareness of young carers in their clinical practice, we were a part of an experience-based co-design process to create a toolkit for healthcare providers to better recognize, engage with, and support young carers in clinical practice. In the following Patient Perspective, we highlight our individual experiences of interacting with the healthcare system as young carers and propose three key recommendations for healthcare systems to better value and integrate young carers. These include the need to recognize that young people can be carers, the importance of support and resources for carers and care recipients alike, and the importance of accessible and reliable primary care.

Imagine you are a healthcare provider—a nurse, physician, or social worker—dropped into the middle of a hospital ward without prior training. Suddenly, you are expected to administer medications, change wound dressings, or coordinate care across providers, despite having no prior experience or knowledge. Although hypothetical, this scenario describes the reality faced by many young people who become caregivers for a family member or friend. Young carers (YCs) take on significant care responsibilities, often without recognition, support, or appropriate training.^1,2^ We use the term young carer to describe caregivers under the age of 25 years.^2^

In Canada, a country of more than 40 million people, over a million young people (ages 15 to 24) are estimated to provide some level of care support.^3^ The experiences of YCs are diverse, spanning genders, cultural backgrounds, medical conditions, disabilities, and the nature of caring relationships. Interactions with the healthcare system are understudied, with previous work identifying challenges such as insufficient communication, lack of collaboration, and difficulties navigating the healthcare system.^4^ We, as YCs and advisors on a previous research and codesign project exploring Canadian healthcare providers’ awareness of YCs, share the following personal narratives and recommendations as a call to action for healthcare providers and health systems to strengthen their engagement and support of YCs in clinical practice.

## Perspectives

**YC1:** What it means to me to be supported as a YC is to be acknowledged by the healthcare system for all the care that I do provide. I am the appointment booker, wound care helper, food feeder, clothes changer, poop wiper, blood sugar checker, insulin giver, bather, exerciser, translator, advocate, resource finder, and so much more. I help with all aspects of daily care activities and assist nurses, doctors, and other healthcare workers when they ask questions about my loved one's care needs. I do this all while simultaneously working a full-time job and having to balance my academics in school. I want to feel celebrated, recognized, seen, and heard. It means so much to me when a healthcare professional, such as a nurse or doctor, compliments me and says that I am doing a good job taking care of my loved one. This simple recognition makes me know that I am cared for and appreciated, too.

**YC2:** Caring for the caregiver—something that is often overlooked. Adolescence is meant to be a time to grow, explore, and build healthy habits. But as a young caregiver, I often felt like I couldn’t just be a kid. I was managing medications, offering emotional support, and navigating grief without knowing how to cope. The stress built up until I burned out and had to seek help. That's why physicians and allied healthcare professionals need to understand how caregiving impacts youth, especially without proper support. Instead of letting stress pile up, we need to intervene early and ensure young caregivers get the help they need. This means early identification, regular emotional check-ins, and access to resources that help them cope and thrive.

**YC3:** I never know what will happen next, and that makes my stomach constrict. Being a YC isn’t just about tending to someone's physical needs—it also means acting as a mediator, translator, healthcare liaison, and filling the endless combinations of roles that are usually reserved for trained professionals, all to ensure your loved one's survival. Tomorrow, I might have to be a personal support worker; then, a few days later, a crisis intervention specialist with detailed knowledge of how to navigate police, psychiatric hospitals, and long-term treatment plans. I am a thinking–feeling young person managing this level of uncertainty. Constriction. When I was attending high school with my severely disabled mother, who needed a care home but couldn’t find or afford one, what should I have chosen: my mom, my home, or my education? That's the kind of impossible situation many YCs such as me face every day. Care relations are connective; the constriction of one partner is always felt by the other partner. If care is truly the goal, then recognizing the shifting roles and pressures placed on YCs is essential—not just to acknowledge their experience, but to begin loosening the grip of the ongoing tension so they aren’t left carrying long-term harms in their own bodies and futures.

**YC4:** Caring for my mother since I was 16, after she developed schizophrenia, meant navigating the healthcare system alone—without support, guidance, or even acknowledgment. For 7 years, I tried and failed to access services, which left me completely responsible for her care at home. I constantly feared child protective services would step in; while home wasn’t ideal, I worried that the alternative would be even more traumatic. When my mother was finally hospitalized, I was left out of her treatment planning because staff assumed I wasn't involved—just because I was a teenager.

No one ever asked me who I was caring for, what I needed, or how I was coping. I wasn't identified as a young caregiver, nor referred to any support programs. Even a single question—“Are you supporting someone at home?”—could have changed everything. I want clinicians to know that we exist, and that we often carry these roles invisibly. Acknowledge us. Ask about our responsibilities.

I didn’t fully understand the weight I was carrying until years later. But I know this: when healthcare professionals see and support young caregivers, it relieves some of that weight—and improves care for the person we’re looking after, too. Recognition is more than kindness. It's a form of intervention.

**YC5:** I grew up as a YC, not realizing I was one. How could I have known that my experience could be described so simply, in 2 short words? Caregiving at a young age is as formative as it is confusing. Are we not meant to be the ones being cared for? Discovering the term “young carer” only a few years ago helped me understand my experience and appreciate the richness that caregiving brought to my life. To me, caregiving means committing to a loved one, overcoming life's challenges together. It is a thankless choice we make, not for lack of gratitude, but because it is only natural. I often wonder how things might have been different had my role been recognized earlier. Maybe I wouldn’t have felt as confused, worried, and isolated. It might have opened the door to better emotional and practical support for my family and me. Recognizing YCs goes beyond validation: it means empowering them to care for a loved one without losing themselves in the process. Empowering them to realize the profound value of the care they exchange with a loved one.

## Recommendations

Through writing these narratives, we aim to articulate an understanding of how challenges compound, without support, for a YC who interacts with the healthcare system. The nature of each relationship is unique and reciprocal. As we identify recommendations for healthcare providers to better support YCs, we underscore the nuance and complexities that exist within caring relationships.

### 1. You Need to see us to Support us

YCs are often overlooked in healthcare settings, largely because their age masks the multitude of roles they play. This lack of recognition can leave YCs without the support they need and deserve. To truly support YCs, healthcare providers must first see them^4^: recognize the invaluable contributions they make, the position they hold, and the challenges they face. YCs have valuable knowledge and expertise to offer healthcare teams, and partnering with YCs can help to provide better patient-centered care.

### 2. Constraint Impacts Everyone in the Caring Relationship

A holistic view of caring recognizes that without adequate support, every person in the family unit can struggle with care responsibilities. Inadequate access to healthcare leaves YCs overwhelmed and overburdened, especially when they may not be equipped to take on informal labor. While individual support for YCs, such as peer or informational support, *can help to reduce caregiver burden*, YCs also highlight the need for the care recipient to receive adequate support themselves.^5^ Healthcare providers play a crucial role in advocating for enhanced respite services, home and community care, and adequate support for the family unit. Addressing these needs can foster a healthier, more enduring, and balanced caring environment for all.

### 3. True Support Comes From a Reliable, Accessible, and Integrated Health System

Family doctors are uniquely positioned to provide longitudinal support and act as champions for YCs as they interact with various parts of the healthcare system.^6^ However, over 6 million Canadians do not have access to a family doctor, creating significant challenges for healthcare providers, patients, and YCs alike.^7^ For YCs, this often means taking on additional caregiving responsibilities, such as coordinating care between providers, and managing medications at home.

To effectively support YCs, all providers have a role to play in initiating dialogue about both the care recipient's and YC's support needs. In addition to acknowledging their role, this allows for the identification and documentation of areas in which YCs may require ongoing educational or community-based support. By approaching conversations about support needs with sensitivity to the personal, familial, and cultural values that can shape caregiving, providers can understand how to best support their patients and YCs along their journeys.

## Conclusion

Similar to carers of all ages, YCs are left to pick up the pieces when the person they care for does not have enough support. This underrepresented demographic of carers faces a distinct set of challenges, as caring without adequate support may contribute to mental, physical, emotional, financial, and social challenges during key periods of development.^8–10^ Providers should be aware that young people can be caregivers, and that they often play an underappreciated, yet critical role in the health of their patients. Acknowledging YCs as members of the healthcare team recognizes the importance of their role and should be emphasized as part of patient- and family-centered care.

Individual-level resources for YCs can help to address some needs, but a truly supportive healthcare system requires access to integrated primary, home, and community care. Ensuring meaningful support for YCs and care recipients demands time, effort, and energy from healthcare providers, but these investments will provide benefits to patients, families, and the healthcare system as a whole.
